# Validation of genotype cluster investigations for *Mycobacterium tuberculosis*: application results for 44 clusters from four heterogeneous United States jurisdictions

**DOI:** 10.1186/s12879-016-1937-9

**Published:** 2016-10-21

**Authors:** Larry D. Teeter, Padmaja Vempaty, Duc T. M. Nguyen, Jane Tapia, Sharon Sharnprapai, Smita Ghosh, J. Steven Kammerer, Roque Miramontes, Wendy A. Cronin, Edward A. Graviss, on behalf of the Tuberculosis Epidemiologic Studies Consortium

**Affiliations:** 1 Houston Methodist Research Institute, Houston, TX USA; 2 Centers for Disease Control and Prevention, Atlanta, GA USA; 3 Emory School of Medicine, Atlanta, GA USA; 4 Massachusetts Department of Public Health, Jamaica Plain, MA USA; 5 Northrop Grumman Corporation, Centers for Disease Control and Prevention Programs, Atlanta, GA USA; 6 Maryland Department of Health and Mental Hygiene, Baltimore, MD USA; 7 Present Address: Texas Department of State Health Services, HSR 6/5, South Houston, TX USA; 8 Department of Pathology and Genomic Medicine, Houston Methodist Research Institute, Mail Station: R6-414, 6670 Bertner, Houston, TX 77030 USA

**Keywords:** Tuberculosis, Epidemiology, Genotype, Cluster investigation, MIRU-VNTR, Spoligotype, Contact investigation, Surveillance

## Abstract

**Background:**

Tracking the dissemination of specific *Mycobacterium tuberculosis* (*Mtb*) strains using genotyped *Mtb* isolates from tuberculosis patients is a routine public health practice in the United States. The present study proposes a standardized cluster investigation method to identify epidemiologic-linked patients in *Mtb* genotype clusters. The study also attempts to determine the proportion of epidemiologic-linked patients the proposed method would identify beyond the outcome of the conventional contact investigation.

**Methods:**

The study population included *Mtb* culture positive patients from Georgia, Maryland, Massachusetts and Houston, Texas. *Mtb* isolates were genotyped by CDC’s National TB Genotyping Service (NTGS) from January 2006 to October 2010. *Mtb* cluster investigations (CLIs) were conducted for patients whose isolates matched exactly by spoligotyping and 12-locus MIRU-VNTR. CLIs were carried out in four sequential steps: (1) Public Health Worker (PHW) Interview, (2) Contact Investigation (CI) Evaluation, (3) Public Health Records Review, and (4) CLI TB Patient Interviews. Comparison between patients whose links were identified through the study’s CLI interviews (Step 4) and patients whose links were identified earlier in CLI (Steps 1–3) was conducted using logistic regression.

**Results:**

Forty-four clusters were randomly selected from the four study sites (401 patients in total). Epidemiologic links were identified for 189/401 (47 %) study patients in a total of 201 linked patient-pairs. The numbers of linked patients identified in each CLI steps were: Step 1 - 105/401 (26.2 %), Step 2 - 15/388 (3.9 %), Step 3 - 41/281 (14.6 %), and Step 4 - 28/119 (30 %). Among the 189 linked patients, 28 (14.8 %) were not identified in previous CI. No epidemiologic links were identified in 13/44 (30 %) clusters.

**Conclusions:**

We validated a standardized and practical method to systematically identify epidemiologic links among patients in *Mtb* genotype clusters, which can be integrated into the TB control and prevention programs in public health settings. The CLI interview identified additional epidemiologic links that were not identified in previous CI. One-third of the clusters showed no epidemiologic links despite being extensively investigated, suggesting that some improvement in the interviewing methods is still needed.

**Electronic supplementary material:**

The online version of this article (doi:10.1186/s12879-016-1937-9) contains supplementary material, which is available to authorized users.

## Background

Tuberculosis (TB) contact investigation (CI) is a disease control strategy that performs a crucial role in understanding the most relevant epidemiologic factors influencing TB transmission between individuals [1]. In addition to CI, tracking the dissemination of specific *Mycobacterium tuberculosis* (*Mtb*) strains in populations is an important tool used to understand TB transmission dynamics [2]. For over 20 years, investigators have been discovering and utilizing genetic elements of the *Mtb* genome as molecular genotype markers [3]. The *Mtb* genotyping methodologies include utilizing the direct repeat locus-based spacer oligonucleotide typing (spoligotyping) [4, 5] and mycobacterial interspersed repetitive unit-variable number of tandem repeat (MIRU-VNTR) typing [6]. These genotyping techniques have been routinely used by the Centers for Disease Control and Prevention (CDC) since 2004 [7].

United States (US) public health departments evaluate persons having known contact with infectious TB patients to identify and treat individuals for whom TB transmission results in active TB disease or latent TB infection (LTBI). Because of the difficulty in identifying and assessing all individuals potentially infected by a given TB patient, CIs provide an incomplete picture of TB transmission. The investigation of TB transmission has been enhanced with the application of *Mtb* genotyping [8]. When *Mtb* genotyping is conducted routinely on all or nearly all *Mtb* isolates from a given jurisdiction, persons with isolates that have the same genotype are termed “clustered” and are suspected of being transmitted recently. Individuals with *Mtb* isolates that have unique genotypes are termed “non-clustered”. TB development in these persons is considered to be due to reactivation of previously acquired LTBI, recent transmission with someone who was not genotyped, transmission from a person outside the 3-year surveillance time window or geographic area, or relapse of a prior episode of TB disease [9].

Genotypic data can facilitate the detection of previously unsuspected transmission [10–12]. Furthermore, when TB patients are identified as epidemiologic-linked through CI, TB transmission can be confirmed or refuted by matching (concordant) or discrepant (discordant) genotypes, respectively [13]. Due to issues concerning the discriminatory power of the genotyping techniques used [8, 14, 15], as well as the endemic level of genotype in a jurisdiction [16], it cannot be assumed that TB patients with matching genotypes result from the same chain of transmission. However, transmission between TB patients with matching genotypes can be verified by detecting epidemiologic linkages, which include: timing, interactions, or relationships among the persons [17]. Epidemiologic investigations of TB patients having genotypically matched *Mtb* isolates can uncover transmission venues and epidemiologic links between persons not identified by routine CI [11]. Public health investigators refer to these additional efforts as cluster investigations (CLI). The current study implements a standardized process for conducting CLI systematically and validates the application of this process to a set of randomly selected *Mtb* clusters identified in public health settings.

## Methods

### Population

*Mtb* culture-positive patients from four study sites reported to the CDC from January 2006 to October 2010, whose *Mtb* isolates were genotyped by CDC’s National TB Genotyping Service (NTGS), were evaluated for *Mtb* clustering. Study sites, Georgia (GA), Maryland (MD), Massachusetts (MA) and Houston (HOU), Texas, were members of the Tuberculosis Epidemiologic Studies Consortium, a consortium of US sites funded by CDC to conduct TB epidemiologic research [18]. All sites except Texas evaluated TB patients for *Mtb* clustering in counties throughout the state. In Texas, only TB patients reported in the City of Houston jurisdiction (HOU) were evaluated. The study was approved by Institutional Review Boards at CDC and each study site.

### TB cluster selection process

*Mtb* isolates from all patients were characterized by NTGS using spoligotyping and 12-locus MIRU-VNTR (MIRU12). Each unique combination of spoligotype and MIRU12 results is assigned a “PCRType” [19]. Clusters were defined as two or more TB patients with the same PCRType in a given public health jurisdiction (county or HOU) during the study period. Clusters were eligible for random sampling selection if the cluster consisted of at least three TB patients residing in the same given public health jurisdiction, whose TB status were reported between January 1, 2006 and the time of cluster evaluation. Eligible clusters for each of the four sites (see above) were assigned to three priority groups (low, medium, and high priority) based on their calculated log-likelihood ratio (LLR: <1.00, 1.00-5.79, and ≥5.80, respectively) associated with the public health cluster priorities [20, 21]. After reviewing the geospatial scores [21], initial expert panel rankings, and cluster investigation findings, the CDC statistician and the expert panel determined the log-likelihood ratio (LLR) cut-points that were associated with high-, medium-, and low-priority clusters in our surveillance data. Clusters were then randomly selected from each group. In total, 44 clusters (11 per site) were selected for further investigation. Details of sample size considerations and the sample selection strategy are provided in Additional file 1.

### Epidemiologic links

An epidemiologic link was defined as relationships between two TB cases within a cluster who were determined to have likely shared air space while at least one of the cases had active TB disease. Epidemiologic links were considered **definite** if two cases named each other as a contact or were identified as having been in the same place at the exact same time; **probable** if the cases were in same place in the same timeframe (same week); and **possible** if the cases were in the same place possibly at the same time (month or season). A homogeneous attribute was defined as a single epidemiologic characteristic describing all patients in a given cluster.

### Cluster investigation

Beginning in late 2009, TB surveillance data for all subjects were obtained using the Report of Verified Case of Tuberculosis (RVCT) [22] through collaborations with local public health staff. The subjects were part of the selected clusters in the data routinely collected by the CDC’s National Tuberculosis Surveillance System.

**Fig. 1:**
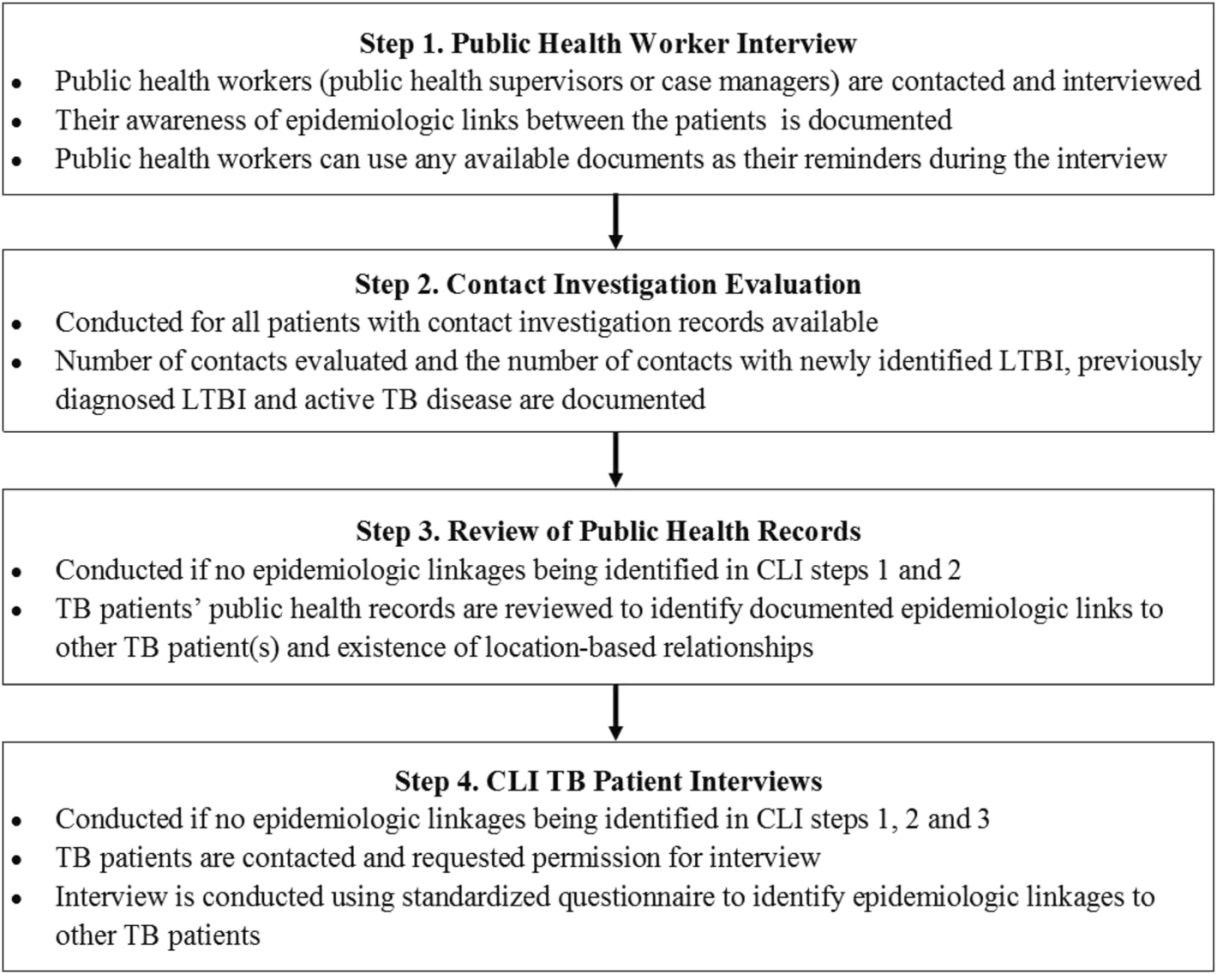
Steps for cluster investigation

In coordination with local TB programs, CLIs for each selected cluster were conducted to determine whether TB patients in a given cluster had epidemiologic-linkages. Patients in selected clusters who were identified after cluster selection occurred were also investigated. A study protocol was developed whereby CLIs were carried out in a stepwise fashion (Fig. 1 and Additional file 2-CLI Instruments):

- **Step 1: Public health worker interview**
  Public health workers (public health supervisors, case managers, disease intervention specialists or contact investigators) for each clustered TB patient were contacted by study staff and asked whether they were aware of any epidemiologic links between the patients. If epidemiologic links were identified between two or more patients in the cluster, that information was documented. The public health workers could use any available documents as mental reminders during the interview.
- **Step 2:** **Contact investigation evaluation**
  In coordination with local TB program staff, contact investigation records of patients in clusters were collected and reviewed to determine whether epidemiologic linkages to other TB patients were identified during the routine CI. For each patient evaluated, the number of contacts evaluated and the number of contacts with newly identified LTBI, previously diagnosed LTBI and active TB disease were documented.
  Public health worker interviews and contact investigation evaluations were carried out on each study patient except when no public health worker could be contacted or when contact investigations were not done. After each single epidemiologic link was established, investigations routinely continued to explore additional epidemiologic links between a patient and other patients in a given cluster.v
- **Step 3: Review of public health records**
  If no epidemiologic links were identified in Steps 1 and 2, TB patients’ public health records which contain documentation of any intake or follow-up patient interviews conducted by the health department were reviewed to determine whether there were documented epidemiologic links to other TB patient(s), and whether location-based relationships existed between patients in the same cluster (e.g. residential, social, or medical settings).
- **Step 4: CLI TB patient interviews**
  If no epidemiologic links were identified between patients in a cluster from CLI Steps 1–3, patients were contacted and interviewed after verbal consent was obtained using a pilot-tested interview instrument (Additional file 2), beginning with the most recently diagnosed subject. The interview instrument was designed to facilitate identification of epidemiologic linkages to other TB patients. For every epidemiologic link identified, estimated dates of symptom onset, relationship between patients, the most frequent patient-pair setting where transmission may have occurred, and the CLI step where the link was identified were documented.
  Epidemiologic links were investigated only if both patient-isolates were genotyped. CLI study instruments (Additional file 2) contained items designed to collect details of the study patients’ frequently visited locations, which could be evaluated as possible venues for transmission.

### Data management and analysis

Study data were entered into a Microsoft Access 2003 (Redmond, WA) database by site staff and merged for analysis by the data coordinating center at the Texas site. National summary data on study PCRTypes (number of patients and the number of states reporting the given genotype) were provided by the CDC. To summarize the characteristics of study clusters, patients in a given cluster were compared to all other study patients by select demographic and behavioral characteristics and two-sided *P*-values were calculated. Clusters associated with at least one epidemiologic link were compared to those without identified epidemiologic link by demographic, behavioral, clinical and genotypic variables.

Comparison between patients whose linkage was identified through the study’s CLI interviews (Step 4) and patients whose linkage was identified earlier in CLI (Steps 1–3) was conducted using univariate and multiple logistic regression. Statistical analyses were conducted using Stata/SE 13.1 (StataCorp LP, College Station, TX).

## Results

From 2006 to 2010, there were 62,642 reported TB cases in the US, reflecting a TB case rate of 4.1 cases/100,000 [23]. During the same time period, study site jurisdictions reported the following number of TB cases: MD-1239, MA-1208, GA-2291 and HOU-1315, corresponding to an average TB rate of 4.4, 3.7, 4.8 and 12.5 cases/100,000, respectively [23]. The proportion of *Mtb* culture-positive patients that were genotyped during the study period was 82.1 % for the US and 98.5, 89.9, 84.4 and 85.8 % for MD, MA, GA and HOU, respectively [19]. From a pool of 132 eligible clusters (MD-25, MA-23, GA-35, HOU-49), 44 clusters (11 clusters from each site) corresponding to 38 distinct PCRTypes were randomly selected for investigation. Three PCRTypes (PCR00002, PCR00016 and PCR00017) were investigated in more than one study jurisdiction (Table 1). Most of the PCRTypes were of Euro-American (L4) or East Asian (L2) lineage (*n* = 29 and *n* = 7, respectively), but one PCRType each was identified of East African Indian (L3) and Indo-Oceanic (L1) lineages. PCRTypes found in the study were also seen nationally with a distribution range from one to 46 states. Three PCRTypes were seen in no US state other than that associated with the study site during the study period: PCR06732 (GA), PCR04837 (TX) and PCR04846 (TX).

**Table 1:** Study genotypes

PCRType	Cluster(s)	Jurisdictions*	Lineage†	Spoligotype	MIRU12	Study cases	US cases‡	States with genotype‡
PCR00002	05, 21, 32, 35, 38	5 (GA/MA3/MD)	L2	000000000003771	223325173533	44	1619	46
PCR00015	44	1 (GA)	L4	777776777760601	224325153323	15	749	43
PCR00016	16, 36	2 (GA/MA)	L4	700036777760731	222325143223	29	227	30
PCR00017	25, 43	2 (GA/TX)	L4	777776777760601	224325153324	7	139	28
PCR00022	22	1 (MA)	L4	777777777720771	225325153323	3	435	42
PCR00036	41	1 (TX)	L2	000000000003771	223425173563	8	165	22
PCR00041	28	1 (TX)	L1	677777477413771	254326223432	13	1392	46
PCR00044	17	1 (MD)	L3	703377400001771	227425113434	5	110	26
PCR00051	39	1 (TX)	L4	776037777760771	223125163324	29	164	28
PCR00079	03	1 (MD)	L4	777777777760771	223125153324	5	149	30
PCR00169	18	1 (MD)	L4	777776777760771	224325124324	4	41	17
PCR00224	11	1 (TX)	L2	000000000003771	223325163333	33	116	16
PCR00293	30	1 (GA)	L4	777777777760771	223225141324	16	30	3
PCR00497	26	1 (TX)	L4	776377777760771	233325153324	10	79	23
PCR00578	13	1 (GA)	L4	777776777760771	125325143224	13	43	7
PCR00719	10	1 (TX)	L4	777777777760771	223425143322	15	124	23
PCR00724	06	1 (MA)	L4	777777777760771	225125113322	3	89	24
PCR00849	23	1 (MA)	L4	777777347760471	224315143324	8	39	12
PCR01017	34	1 (MD)	L4	776177607760771	224326133324	4	42	17
PCR01034	37	1 (MA)	L4	777777777760731	223425123324	3	11	5
PCR01046	07	1 (MA)	L4	677777607760771	223226153321	4	58	19
PCR01047	01	1 (MD)	L4	777776777760601	223325153322	16	29	5
PCR01201	12	1 (TX)	L2	000000000003771	223325173534	32	164	25
PCR01571	31	1 (GA)	L2	000000000003771	223225173433	8	32	11
PCR01674	02	1 (MD)	L4	700076777760771	225225153326	6	12	3
PCR01872	15	1 (GA)	L4	777776777760601	223325153324	3	6	4
PCR01873	40	1 (TX)	L4	777776777760601	224325153321	3	28	10
PCR02143	04	1 (MD)	L4	777777777720771	225325153322	3	34	16
PCR02397	29	1 (GA)	L4	777776777760601	223325153323	11	50	10
PCR02651	19	1 (MD)	L4	777777743760771	223215153324	3	15	9
PCR03405	24	1 (MA)	L4	776377777760771	232325143324	9	11	2
PCR03412	08	1 (MA)	L2	000000000003771	222315173543	6	16	5
PCR03588	42	1 (GA)	L4	037776777760601	224325153423	6	17	3
PCR03994	20	1 (MD)	L4	777777600060771	224226123311	3	12	2
PCR04200	33	1 (MD)	L4	776177400000171	223326133323	4	10	3
PCR04837	27	1 (TX)	L4	777776777760601	224315163323	4	5	1
PCR04846	09	1 (TX)	L2	000000000003771	223425163333	7	17	1
PCR06732	14	1 (GA)	L4	777760377760771	223225153325	6	8	1

*Number of study jurisdictions where the genotype was investigated

†L1: Indo-Oceanic; L2: East Asian; L3: East African Indian; L4: Euro-American; ‡Verified cases of tuberculosis reported by CDC, 2006-2010. MIRU: mycobacterial interspersed repetitive units; PCR: polymerase chain reaction

A total of 401 study patients in the 44 selected clusters were evaluated by the CLI method. Median cluster size was six (range 3–33); HOU clusters tended to be larger than those from other sites (median 10 vs. 6, *p* = 0.024). Nineteen clusters (43 %) had only US-born patients and eight clusters (18 %) contained only foreign-born patients (Table 2). Certain single epidemiologic profiles describing all patients in a given cluster were identified for specific clusters (Table 2, “Homogeneous attribute” column).

**Table 2:** Characteristics of study clusters

Cluster	PCRType	Site	Cluster size	US-born	Male	Race^a^	Home-less	Epi-linked^b^	Homogeneous attribute
01	PCR01047	MD	16	94 %	94 %	100 % B	75 %	81 %	Blacks (15/16 US-born)
02	PCR01674	MD	6	100 %	50 %	100 % B	0 %	50 %	US-born Blacks
03	PCR00079	MD	5	100 %	40 %	100 % B	20 %	40 %	US-born Blacks
04	PCR02143	MD	3	100 %	67 %	100 % B	0 %	67 %	US-born Blacks
05	PCR00002	MA	10	20 %	90 %	90 % A	10 %	0 %	none
06	PCR00724	MA	3	0 %	67 %	67 % B	0 %	0 %	Foreign-born (but country mixture)
07	PCR01046	MA	4	25 %	50 %	75 % B	25 %	25 %	none
08	PCR03412	MA	6	100 %	100 %	67 % B	17 %	83 %	US-born males
09	PCR04846	TX	7	100 %	57 %	100 % B	14 %	0 %	US-born Blacks
10	PCR00719	TX	15	53 %	67 %	80 % H	7 %	47 %	none
11	PCR00224	TX	33	100 %	85 %	58 % B	24 %	52 %	US-born
12	PCR01201	TX	32	94 %	88 %	47 % B	44 %	44 %	none
13	PCR00578	GA	13	100 %	77 %	92 % B	31 %	77 %	US-born (12/13 Black)
14	PCR06732	GA	6	50 %	67 %	67 % H	0 %	83 %	none
15	PCR01872	GA	3	100 %	33 %	100 % B	0 %	0 %	US-born Blacks
16	PCR00016	GA	19	95 %	68 %	95 % B	37 %	53 %	18/19 US-born Blacks
17	PCR00044	MD	5	0 %	40 %	100 % B	0 %	60 %	Africa-born Blacks
18	PCR00169	MD	4	50 %	25 %	100 % B	0 %	0 %	Blacks
19	PCR02651	MD	3	0 %	0 %	100 % B	33 %	33 %	Cameroon-born Black females
20	PCR03994	MD	3	100 %	33 %	100 % B	0 %	100 %	US-born Blacks
21	PCR00002	MA	4	25 %	75 %	75 % A	25 %	0 %	none
22	PCR00022	MA	3	0 %	33 %	67 % H	0 %	0 %	Foreign-born (but country mixture)
23	PCR00849	MA	8	88 %	100 %	63 % W	88 %	63 %	Males (7/8 US-born, homeless)
24	PCR03405	MA	9	11 %	89 %	100 % W	11 %	78 %	White (8/9 Portugal-born)
25	PCR00017	TX	3	100 %	67 %	67 % B	33 %	0 %	US-born
26	PCR00497	TX	10	100 %	50 %	100 % B	10 %	20 %	US-born Blacks
27	PCR04837	TX	4	100 %	75 %	75 % B	0 %	0 %	US-born
28	PCR00041	TX	13	31 %	69 %	69 % H	0 %	31 %	none
29	PCR02397	GA	11	100 %	91 %	100 % B	18 %	64 %	US-born Blacks
30	PCR00293	GA	16	100 %	56 %	100 % B	44 %	56 %	US-born Blacks
31	PCR01571	GA	8	25 %	38 %	88 % A	0 %	88 %	none
32	PCR00002	GA	5	0 %	40 %	100 % A	0 %	60 %	Foreign-born Asians (but country mixture)
33	PCR04200	MD	4	0 %	50 %	100 % H	0 %	75 %	Mexico-born Hispanics
34	PCR01017	MD	4	0 %	50 %	100 % H	0 %	50 %	Foreign-born Hispanics (but country mixture)
35	PCR00002	MD	12	0 %	67 %	100 % A	0 %	0 %	Foreign-born Asians (but country mixture)
36	PCR00016	MA	10	100 %	100 %	50 % B	100 %	100 %	Homeless US-born males
37	PCR01034	MA	3	33 %	100 %	100 % B	100 %	0 %	Homeless Black males
38	PCR00002	MA	13	8 %	54 %	92 % A	8 %	0 %	none
39	PCR00051	TX	29	72 %	69 %	52 % H	14 %	31 %	none
40	PCR01873	TX	3	100 %	100 %	100 % B	0 %	67 %	US-born Black males
41	PCR00036	TX	8	100 %	100 %	75 % B	13 %	0 %	US-born males
42	PCR03588	GA	6	100 %	100 %	100 % B	33 %	83 %	US-born Black males
43	PCR00017	GA	4	100 %	75 %	100 % B	50 %	50 %	US-born Blacks
44	PCR00015	GA	15	100 %	47 %	87 % B	7 %	40 %	US-born

^a^Most common race/ethnicity: A-Asian, B-Black/African American, H-Hispanic, W-non-Hispanic white/Caucasian

^b^Percent of patients with an epidemiologic link to another patient with a concordant PCRType

**Fig. 2:**
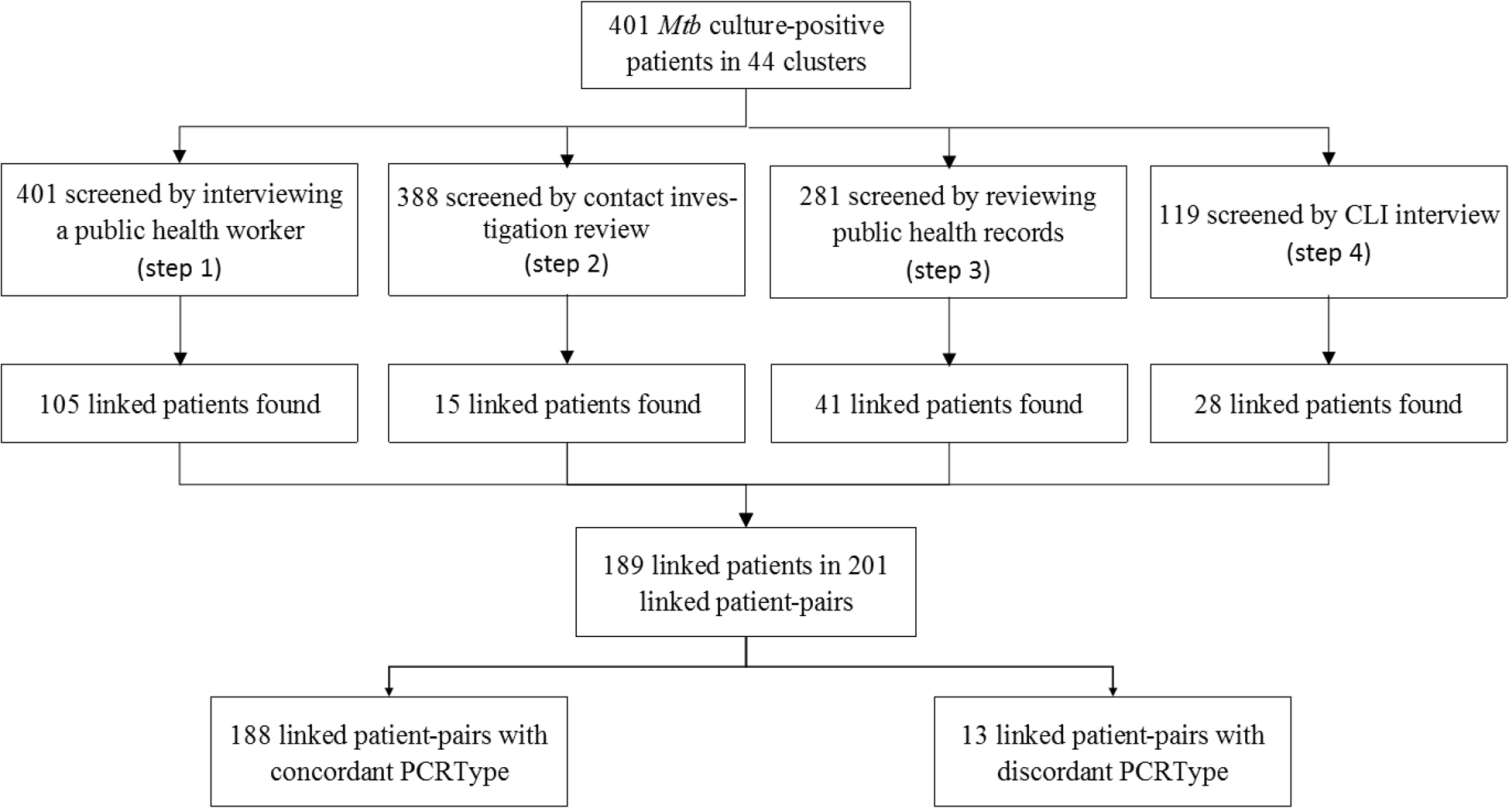
Patient enrollment and study procedures for cluster investigation

In 401 study patients, 189 (47 %) patients were identified with epidemiologic links in a total of 201 linked patient-pairs (Fig. 2), of which 132 (66 %) were definite linkage strength, 27 (13 %) were probable and 42 (21 %) were possible epidemiologic links. Screening by a PHW (Step 1) identified 105/401 (26.2 %) linked patients. Among 388 study patients having contact investigation (CI) records available, CI record review (Step 2) found 15 (3.9 %) linked patients. Patients without CI records (*n* = 63, 16.2 %) were associated with having only extrapulmonary TB manifestations, or being homeless or injection drug users (*p* < 0.05). A total of 3893 contacts were evaluated with a median of five contacts per patient. CI outcomes included 687 (17.6 %) individuals with LTBI, 286 (7.3 %) with prior LTBI, 81 (2.1 %) with active TB disease and the remaining 2839 having no TB history, but associated with the study patient. In reviewing the public health records (Step 3) of patients with no epidemiologic link found in Steps 1–2, 41/281 (14.6 %) linked patients were identified. CLI interviews (Step 4) were completed on 30 % (119/401) of patients with 28/119 (23.5 %) linked patients found (Fig. 2). Among patients who did not have CLI interviews, 27.0 % (76/282) had epidemiologic link(s) that had already been identified through Steps 1–3. The patients had decreased odds for CLI interviews if they were homeless (*p* < 0.001), male (*p* < 0.001) or age 65 or older and increased odds for CLI interviews if they were diagnosed after 2008 (*p* < 0.001) or were from the MD site (*p* = 0.047) (data not shown).

Among 201 linked patient-pairs, 188 (93.5 %) pairs had concordant PCRTypes and 13 (6.5 %) pairs had discordant PCRTypes (66 and 62 % with definite linkage strength, respectively; *p* = 0.7). All of the 13 linked patient-pairs with discordant PCRTypes had discordant MIRU12 patterns (median of three discordant loci), while seven also had discordant spoligotypes. These 13 genotypically discordant, but epidemiologic-linked, patient-pairs were excluded from further consideration because their high level of discordance suggested that the linked patient-pairs were not part of the same transmission chain. The 188 linked patient-pairs with concordant PCRTypes corresponded to only 179 of the 401 study patients (45 %) having epidemiologic links because 75 patients had more than one link identified.

Specific transmission venues were identified for some clusters. Among 19 clusters with at least three pairs of epidemiologic-linked patients (Clusters 01, 02, 08, 10, 11, 12, 13, 14, 16, 20, 23, 24, 28, 29, 30, 31, 36, 39 and 42), 11 (57.9 %) had at least 50 % of their total epidemiologic links associated with a specific venue: four with homeless shelters (Clusters 01, 12, 23 and 36), three with drug houses (Clusters 16, 30 and 42), two with churches (Clusters 14 and 31), one with a bar (Cluster 39) and another with a social club (Cluster 24). Over 90 % of epidemiologic links identified for Clusters 01, 23 and 36 were associated with homeless shelter transmission venues. All epidemiologic links identified for Cluster 42 were associated with a drug house venue and seven of the eight (88 %) epidemiologic links identified for Cluster 24 were associated with a social club transmission venue. Seven (37 %) of the 19 clusters with at least three pairs of epidemiologic-linked patients were mainly (≥50 %) associated with household or non-household close social transmission venue (Clusters 02, 08, 10, 13, 20, 28, and 29). Among 16 epidemiologic linked pairs of the remaining cluster (Cluster 11), seven (44 %) was associated with homeless shelters and four (25 %) was associated with a church.

There was substantial variability by cluster in terms of the proportion of patients with identified epidemiologic links (Table 2, “Epi-linked” column), ranging from 0 to 100 %. No epidemiologic links were identified for patients in 13/44 (30 %) clusters, despite having all four CLI steps completed on 36 % of the 77 patients in these clusters. The number of clusters having all black patients was significantly lower in 13 clusters without epidemiologic links than in those with epidemiologic links [2 (15.4 %) versus 15 (48.4 %), *p* = 0.040]. No difference in the number of clusters having 100 % foreign-born patients was seen between the two groups (data not shown).

Twenty-five percent of epidemiologic links from HOU were identified through CLI. Meanwhile, epidemiologic links from MA and MD had lower odds of being identified by CLI TB patient interviews than linkages from other sites (Table 3; *p* = 0.004 and *p* = 0.036, respectively). All epidemiologic links with a household transmission setting and/or involving relatives were identified earlier than Step 4, while workplace and church transmission settings were associated with identification through CLI TB patient interviews in Step 4 (*p* = 0.032 and *p* = 0.046, respectively). Epidemiologic links involving a black TB patient had higher odds of being identified by early investigation steps (*p* = 0.036). Epidemiologic links including Asians or patients with extrapulmonary TB were associated with identification through CLI TB patient interviews in univariate analysis (*p* < 0.001 and *p* = 0.033, respectively); these associations became non-significant in multivariate results. Definite (strength) epidemiologic links had decreased odds for identification through interviews (*p* < 0.007) (Table 3). All epidemiologic links identified for clusters 14, 19, and 43 were identified by CLI TB patient interviews and over 50 % of links identified for clusters 31 and 39 were identified by CLI TB patient interviews (Data not shown).

**Table 3:** Characteristics associated with epidemiologic links being identified by CLI TB patient interviews (Step 4) versus earlier in CLI (Steps 1–3)

	Epi-linked patient-pairs with concordant PCRTypes		Unadjusted			Adjusted^a^		
	Identified without CLI TB patient interviews (*n* = 158)	Identified by CLI TB patient interviews (*n* = 30)	OR	*p*-value	95 % CI	OR	*p*-value	95 % CI
Study site, n (%)								
Texas (City of Houston)	45 (28.5 %)	15 (50.0 %)	2.51	0.226	0.57, 11.16	0.20	0.191	0.02, 2.25
Massachusetts	26 (16.5 %)	2 (6.7 %)	0.36	0.218	0.07, 1.82	0.04	0.004	0.00, 0.35
Maryland	48 (30.4 %)	1 (3.3 %)	0.08	0.050	0.01, 1.00	0.01	0.036	0.00, 0.73
Georgia	39 (24.7 %)	12 (40.0 %)	2.03	0.357	0.45, 9.22	--		
Race/ethnicity, n (%)								
Asian	4 (2.5 %)	5 (16.7 %)	7.70	<0.001	3.12, 19.00	0.28	0.349	0.02, 4.07
Black	93 (58.9 %)	6 (20.0 %)	0.18	0.010	0.05, 0.66	0.21	0.036	0.05, 0.90
Hispanic	32 (20.1 %)	13 (43.3 %)	3.01	0.211	0.53, 16.97	--		
White	24 (10.8 %)	6 (10.0 %)	1.11	0.889	0.25, 4.94	--		
Transmission setting, n (%)								
Household	29 (18.4 %)	0 (0.0 %)		NA		--		
Close social	27 (17.1 %)	2 (6.7 %)	0.35	0.147	0.08, 1.45	--		
Workplace	5 (3.2 %)	4 (13.3 %)	4.71	0.015	1.36, 16.35	7.16	0.032	1.19, 43.08
Shelter	58 (36.7 %)	4 (13.3 %)	0.27	0.139	0.05, 1.54	--		
Church	5 (3.2 %)	8 (26.7 %)	11.13	0.051	0.99, 125.43	10.98	0.046	1.04, 116.08
Drug house	17 (10.8 %)	2 (6.7 %)	0.59	0.579	0.09, 3.76	--		
Other	17 (10.8 %)	10 (33.3 %)	0.24	0.047	0.06, 0.98	0.25	0.024	0.08, 0.83
Relationship between case pairs, n (%)								
Relative	25 (15.8 %)	0 (0.0 %)		NA		--		
Friend	41 (26.0 %)	4 (13.3 %)	0.44	0.204	0.12, 1.56	--		
Coworker	4 (2.5 %)	4 (13.3 %)	5.92	0.008	1.58, 22.27	--		
Genotype lineage, n (%)								
East Asian (L2) lineage	30 (19.0 %)	12 (40.0 %)	2.84	0.118	0.77, 10.54	0.98	0.976	0.19, 5.07
Euro-American (L4) lineage	122 (77.2 %)	18 (60.0 %)	0.44	0.229	0.12, 1.67	--		
Common genotype in US†, n (%)	8 (5.1 %)	2 (6.7 %)	1.34	0.709	0.29, 6.21	--		
Extra-pulmonary only site of TB, n (%)	8 (5.1 %)	6 (20.0 %)	4.69	0.033	1.13, 19.42	9.54	0.105	0.62, 146.32
US-birth, n (%)	132 (83.5 %)	24 (80.0 %)	0.79	0.739	0.19, 3.21	--		
HIV infection, n (%)	54 (34.2 %)	6 (20.0 %)	0.48	0.301	0.12, 1.92	--		
Incarcerated at TB diagnosis, n (%)	10 (6.3 %)	1 (3.3 %)	0.51	0.567	0.05, 5.10	--		
Homeless, n (%)	83 (52.5 %)	8 (26.7 %)	0.33	0.136	0.08, 1.42	--		
Excess alcohol use, n (%)	112 (70.9 %)	15 (50.0 %)	0.41	0.146	0.12, 1.37	--		
Non-injection drug use, n (%)	70 (44.3 %)	8 (26.7 %)	0.46	0.058	0.20, 1.03	0.48	0.118	0.19, 1.21
Patient pairs with different zip codes in same jurisdiction, n (%)	99 (66.9 %)	22 (88.0 %)	3.63	0.022	1.21, 10.89	2.84	0.174	0.63, 12.83
Patient pairs from different jurisdictions, n (%)	10 (6.3 %)	5 (16.7 %)	2.96	0.103	0.81, 10.89	4.41	0.070	0.89, 21.95
Epidemiologic link strength = “definite”, n (%)	114 (72.2 %)	10 (33.3 %)	0.19	<0.001	0.08, 0.45	0.22	0.007	0.22, 43.23

*OR* odds ratio, *CI* confidence interval, *CLI* cluster investigation

^a^Adjusted in the multivariate model. The final model included study site, race/ethnicity, transmission setting, genotype lineage, TB site, non-injection drug use, pairs with different zip codes in same jurisdiction, pairs from different jurisdictions and definite epi-link link strength

†PCRTypes associated with > 400 TB patients in the U.S. 2006-2010 and reported in > 40 states (PCR00002, PCR00015, PCR00022, PCR00041)

## Discussion

Contact investigation of individuals who had contact with TB patients is a cornerstone of public health TB control [1]. However, limitations of the concentric circle approach to contact investigations have been highlighted by reports of TB transmission not found through traditional contact investigation methods [11, 23–26]. In our study, a considerable number of additional linked patients (*n* = 28; 14.8 % of all identified patients with at least one link to another person in the cluster) found in the CLI interview were not identified through the previous CI (Fig. 2).

Molecular epidemiologic data suggests that routine contact investigations, targeting household, work, and school contacts, commonly miss other locations where infectious TB patients spend time and transmit disease, especially leisure or social settings [11, 17, 27, 28]. The CDC contact investigation guidelines [1] recommend collecting information on potential transmission settings during patient CI interview. In the absence of named TB patient contacts, location-based information on possible transmission venues collected routinely during patient interviews can be useful in establishing relationships between genotypically linked TB patients [11].

By looking for homogeneity within a cluster using routinely collected surveillance data, we were able to generate characteristic profiles for many clusters. These cluster-specific epidemiologic profiles provided hints into potential transmission venue types for given clusters and provided insight into questions to ask, or locations to look for while seeking epidemiologic linkages during CLI steps.

CLI steps were prioritized to minimize resources required to uncover epidemiologic links by first asking health department staff who were directly involved in the TB patient’s care if they were aware of links to other patients (Step 1). When applied in a local health department context, existing knowledge of clusters or patient relationships is available through communication with a case manager, disease intervention specialist, or contact investigator (public health workers). Existing contact investigation records were then reviewed for documented links (Step 2). The next investigation step, entailing review and evaluation of public health records, added a more time-intensive and analytic component to investigations (Step 3). Finally, the most resource intensive step was patient re-interviews (Step 4). The analysis of the CLI step where epidemiologic links were determined (Table 3) demonstrated various scenarios where CLI interviews had added utility compared to earlier investigative steps. Higher odds of epidemiologic links were found in association with workplace, when patient-pairs resided in different zip codes within the same jurisdiction and Asian or African American patients (Table 3). Although we found 11 study participants having unknown epidemiologic links through contact investigation review (Step 2), we do not know how many contacts with active TB had epidemiologic links because a contact with active TB might be involved in more than one epidemiologic link.

Limitations to this study include the possibility of not including all patients in a potential genotype cluster given genotype coverage during the study period (especially for GA and HOU), the inability to locate and obtain consent from patients for re-interviews and exclusion of clinically defined and non-genotyped culture-positive patients with epidemiologic links to patients in study clusters. In addition, the infectious period of each patient was not considered. Although beyond the scope of this study, including non-genotyped patients may show a more complete picture of cluster transmission dynamics. Furthermore, NTGS transitioned from using spoligotype and 12-locus MIRU-VNTR (MIRU12) to spoligotype and 24-locus MIRU-VNTR (MIRU24) in 2009 to increase the discriminatory power of MIRU-VNTR [8, 14, 15]. Since this study was initiated in 2009 and included cases in previous years, cluster definition and selection process had to be defined by spoligotype and MIRU12. Given the variant number of epidemiologic links identified by different study sites, interview style (although standardized) may have played a role in potentially influencing subjective and qualitative outcomes. Despite the variation of results seen between sites, one of our study outcomes was to provide additional high-risk TB contacts identified by CLIs. In resource-limited jurisdictions where local funding and resource may not be enough to launch the cluster investigations, the information of high-risk contacts that were missed by the initial CIs is still helpful for evaluation purposes and to help TB programs improve their conventional CI techniques. Lastly, recall bias could not be ruled out, especially in patients who were diagnosed with TB many years before their cluster investigation interview was conducted.

Public health departments need to develop strategies and focus resources to prioritize and investigate clusters that may be of public health concern. An initial step in these investigations should be to evaluate clusters using readily available data. Many data elements needed to investigate clusters in specific jurisdictions are now available to TB control personnel routinely and electronically through the Tuberculosis Genotyping Information Management System [19]. Additionally, the 2009 expansion of the RVCT includes up to two state case numbers for TB patients epidemiologic-linked to the reported patient [22], so health departments can easily assess clusters for epidemiologic links. If the transmission dynamics are poorly understood and the cluster continues to grow, additional resources should be devoted to the CLI, including abstracting public health records of clustered patients and interviewing the TB patients to find epidemiologic linkages between patients beyond those identified by the health department. As we found in this study, re-interviewing patients in a cluster (Step 4), especially when no epidemiologic links have been identified can facilitate the identification of transmission venues and locations that are crucial in interrupting the ongoing transmission and cluster growth. Further study on improving the interviewing methods may be needed to increase the detection rate of epidemiologic links in *Mtb* genotype clusters. In addition, CI record review (Step 2) found 15 (3.9 %) linked patients exemplifying a need for better tools and trainings for contact investigations, which is an essential component of TB control programs.

Despite the continuing decline in US TB rates leading to a decrease of funding for public health activities for TB control, the elimination goals established in 1989 [29] remain unmet. With the recent leveling rates of TB [30], an interruption of the *Mtb* transmission by implementing the expanded and efficient CIs and CLIs would be critical for the success of TB control and prevention programs in the US.

## Conclusion

We validated a practical method to systematically identify tuberculosis epidemiologic links that can be integrated into routine TB control and prevention programs in public health settings. Re-interviewing patients in a cluster can identify additional epidemiologic links that were not found in the previous CLI steps. Improvement of the interview methods and effective contact investigation trainings may be needed as no epidemiologic links were identified in one-third of the *Mtb* genotype clusters.

## Additional files

Additional file 1: Methods section technical details. (DOCX 18 kb)
Additional file 2: Cluster Investigation Instruments. (DOC 117 kb)

## Abbreviations

CDC: Centers for Disease Control and Prevention
CI: Contact investigation
CLI: Cluster investigation
LTBI: Latent TB infection
MIRU-VNTR: Mycobacterial interspersed repetitive unit-variable number of tandem repeat
*Mtb*: *Mycobacterium tuberculosis*
NTGS: National TB Genotyping Service
PHW: Public Health Worker
RVCT: Report of verified case of tuberculosis
