# Supplementary material for: Validation of genotype cluster investigations for Mycobacterium tuberculosis: application results for 44 clusters from four heterogeneous United States jurisdictions
Source: BMC Infect Dis. 2016 Oct 21;16:594. doi: 10.1186/s12879-016-1937-9 (PMC5075185; doi:10.1186/s12879-016-1937-9)
Supplement: Additional file 1: — Methods section technical details. (DOCX 18 kb) [file 12879_2016_1937_MOESM1_ESM.docx]

**Cluster Selection**

Staff at study sites generated rosters of eligible clusters and submitted them to CDC study staff at three six month intervals: September 2009, April 2010 and October 2010. At each time point, and for each eligible cluster, site staff provided the number of patients with the given PCRType in the jurisdiction by year and the number of patients with other genotypes in that jurisdiction by year. CDC study staff used the data provided by sites to calculate the log likelihood ratio (LLR) for each cluster as described (*20*). The LLR statistic (online Figure) compares the likelihood that a cluster of interest occurred within a defined geographic area (LR_1_) with the likelihood that it occurs in the US outside the defined area (LR_2_). The CDC statistician selected 4 clusters for each site in the first two random selections and 3 at each site for the last selection; a total of 44 clusters (11 per site) were selected for investigation.

Sampling was done separately for each site, using a quota method. Each eligible cluster for a site was sorted into 3 groups (low, medium, and high priority), based on calculated LLR and LLR cut points (LLR: <1.00, 1.00-5.79, and >5.80, respectively) associated with cluster priorities determined from the Washington State investigation [*20*]. We then did a random selection from each group; if no clusters were available in the high priority group for a site, we would then select an additional cluster from the medium priority group.

**Sample Size**

The number of clusters to be investigated was determined by calculating the number needed to detect a statistically significant difference between the mean LLR for the medium- and high-priority groups with 80% power, using a one-way analysis of variance. The total number of clusters was based on an alpha of .05, and an estimated standard deviation for the LLR was calculated using all clusters with at least 3 patients in the national database. A ratio of 3 to 1 between the high and medium priority groups’ average LLRs was used as a conservative assumption to detect significance. Final sample size calculations called for a minimum study sample of 42 clusters, with 12 high, 15 medium, and 15 low priority clusters, based on initial LLR scores. A final sample size of 44 clusters was made to evenly distribute the numbers from each of the 4 sites and to select the high, medium, and low priority clusters so as to have a mix of representative clusters within each site.

Results described in the current manuscript do not include any discussion of LLR utility; results of the analysis of this study sample related to LLR are being detailed in a subsequent manuscript.

**Ethical Considerations**

IRBs at all four study sites approved the protocol either through full review (MD Department of Health and Mental Hygiene IRB and TX Department of State Health Services IRB), by deferring to the findings of the CDC central IRB (MA Department Public Health Human Research Review Committee, Johns Hopkins University School of Medicine IRB [MD site] and Emory University IRB [GA site]), or by determining that the project was non-research and exempt from review due to it being “routine public health practice” (GA State Department Human Resources IRB). IRB approvals at non-TX sites permitted obtaining verbal consent from subjects invited to be interviewed and consent was documented on a patient tracking form. TX Department of State Health Services IRB required written informed consent from all TX study subjects interviewed.
