# Supplementary material for: Validation of genotype cluster investigations for Mycobacterium tuberculosis: application results for 44 clusters from four heterogeneous United States jurisdictions
Source: BMC Infect Dis. 2016 Oct 21;16:594. doi: 10.1186/s12879-016-1937-9 (PMC5075185; doi:10.1186/s12879-016-1937-9)
Supplement: Additional file 2: — Cluster Investigation Instruments. (DOC 117 kb) [file 12879_2016_1937_MOESM2_ESM.doc]

**Step 1: Public health worker interview**

| **Site Name:________________________ RVCT#_________________**  **Patient name:__________________ Date of birth (DOB):_______________**    **Cluster #: ____________________ Date:___________________________**  **Case Manager: __________________ Case Manager form completed Yes/No:___________**    **If “No” give reason: _________________________** |
| --- |

**1. Was a contact investigation done/completed on this patient?**

1- Yes 2- No 3- Unknown

**2. To your knowledge, are there any known relationships/links between this patient and any other TB patients?**

1- Yes 2- No 3-Unknown

**If yes, with whom? _____________________________**

**County/jurisdiction ____________________________**

**3. To your knowledge, what was the symptom onset date for this patient? _____________________________**

**4. To your knowledge, what are the main places that were frequented/ visited by the patient? Where possible, please provide an address or neighborhood.**

1- Restaurants/Pubs ____________________________________________________________

2- Church _____________________________________________________________________

3- Work _______________________________________________________________________

4- Other (Specify :________________________________________________________________)

*For each identified relationship in the cluster, complete the following*:

**5. What relationship(s) does this patient have with the other patient?**  1- Family / Relative 3- Co-worker 5- Other __________________________

2- Friend 4- Fellow student

**6. Who do you think is the likely source patient? (symptom onset date)** _______________

**7. Generally speaking, where do you think the patient seeks care/treatment?**

1-The health dept 3- Hospital

2- Private provider 4- Other _________________________

3- Hospital

**8. Identify the setting where transmission took place.**

- 1. Household 5- Leisure/Social
  2. Congregate setting (example: Homeless shelter.) 6- Unknown
  3. Work 7- Other __________________________
  4. School

**9**. **How did you learn about the relationship/link?**

1 - Contact investigation 3- Genotyping

2 - “Gut feeling” 4- Other _____________________

**10. What else would you like to share about the patient?** ________________________________________________________________________________________________________________________________________________________________________________________________________________________

____________________________________________________________________________________________________________

**Step 2: Contact investigation evaluation (obtain contact list from local health department)**

**Cluster designation (PCRtype):________________ Patient RVCT#:________________ Local ID#:______________________**

**Date Form Completed: ____________ By Whom: ____________________________**

|  | **Total**  **#** | **Previously positive**  **(TST or IGRA)** | | **Contacts to test** | **Contacts Tested** | | **New positive test**  **(TST or IGRA)** | | | **Total positive**  **(Previous + New)**  **(TST or IGRA)** | | | **# of Active cases** |
| --- | --- | --- | --- | --- | --- | --- | --- | --- | --- | --- | --- | --- | --- |
|  |  | **#** | **%** | **#** | **#** | **% of to test** | **#** | **% of tested** | **% of to test** | **#** | **% with result** | **% of total** |  |
| **Close contacts**  **identified** |  |  |  |  |  |  |  |  |  |  |  |  |  |
| **Other contacts identified** |  |  |  |  |  |  |  |  |  |  |  |  |  |

**Details on contact with active TB disease**

|  | **Name** | **Local ID #** | **RVCT #** | **Culture Result** | | | **Relationship to Index Patient, as identified during CI** | **PCR type** | **PCR not available**  **or unknown** |
| --- | --- | --- | --- | --- | --- | --- | --- | --- | --- |
|  |  |  |  | **Unkn** | **Neg** | **Pos** |  |  |  |
| 1 |  |  |  |  |  |  |  |  |  |
| 2 |  |  |  |  |  |  |  |  |  |
| 3 |  |  |  |  |  |  |  |  |  |
| 4 |  |  |  |  |  |  |  |  |  |
| 5 |  |  |  |  |  |  |  |  |  |

**Step 3: Review of public health records**

**Medical Record Data Abstraction Form**

**Name of abstractor _______________________ Date of data abstraction ___________________**

**1) Identifiers**:

1.1. **RVCT #** :

1.2**. Local ID #** ___________________ **Public health, hospital, private clinic, Other** *(circle)*:_______

1.3. **Name:** **Last** ___________ **First:** _____________

1.4. **Alias/Maiden:** **Last** ___________ **First:** _____________

1.5. **DOB:** ____/____/____ (MM/DD/YY) 1.7. **Gender**: M F

1.6**. Address:** (most recent): ______

(Street name) (Apt.#)

______

(Town) (State) (ZIP)

1.7**. Home**  (____) ______ - ______ **Cell**  (____) ______ - _____ **Work** (____) ____ - ____

**Other** (____) ______ - _____

1.8**. Address:** (former): ________

(Street name) (Apt.#)

________

(Town) (State) (ZIP)

1.9. **Primary languages:** (other than English): Specify ____________________________________

**2) Medical Risk:**

2.1 **HIV results (most recent results):**  Positive  Negative  Indeterminate  Refused

 Not offered  Test done, results unknown  Unknown  No documented;

If test results available, date:____________

2.2 **Immunosuppressive conditions (not HIV/AIDS):** Yes  No Unknown

 Not documented

2.2a. If yes, (check all that applies) Cancer  Diabetes Mellitus  End-stage renal failure

Other (Specify): _____________________

2.3 **Immunosuppressive drugs** (specify)

2.4 **Regular visit to clinic or hospital for treatment for immunosuppressive condition**: Yes  No

If yes: name of hospital or clinic: _________________Location: _____________________________

**3) TB History**

3.1  **Reported TB Exposure:**  No known exposure Unknown  Yes  to whom, when and where?

Name : Location:___________________________________

Relationship : Time of Exposure: _____/_____ to _____/_____

mm/yy mm/yy

Name : Location:___________________________________

Relationship : Time of Exposure: _____/_____ to _____/_____

mm/yy mm/yy

3.2**. Tuberculin Skin Test (TST) History: *Start with most recent***

3.2a. Date ____/_____/_____(MM/DD/YY)  “Positive”  “Negative”  Unknown

Results: (mm induration) _____mm  Unknown

3.2b. Date _____/_____/_____ (MM/DD/YY)  “Positive”  “Negative”  Unknown

Results: (mm induration) _____mm  Unknown

3.3**. Interferon Gamma Release Assay (QFT) History: *Start with most recent***

3.3a. Date ____/_____/_____ (MM/DD/YY)

 “Positive”  “Negative” “Indeterminate”  “Unknown”

3.3b. Date ____/_____/_____

 “Positive”  “Negative” “Indeterminate”  “Unknown”

3.4. **Previous TB Disease: (***most recent episode***)**   “Yes”  “No” “Unknown”

3.4a. If yes, date of diagnosis: _____/______ (MM/YY)

3.4b. Site of Disease (check all that apply):  Pulmonary  Extra-pulmonary

3.4c. Laboratory Culture results: Positive Negative Unknown

**4) Current TB diagnosis**

4.1. **Treatment start date (Diagnosis date):** _______/________(MM/YY)

City State

4.2 **Reported Symptoms for current TB diagnosis**: (check all that apply  Cough  Hemoptysis

 Weight loss  Fatigue  Fever  Night sweat  Bone pain  Other Specify __________________

4.3 **Reported symptom onset date:** _____________________ (MM/YY)

4.4. **Major site of disease**: (check all that apply)

 Pulmonary  Laryngeal  Extra-pulmonary

4.5. **CXR at diagnosis**: Date ___/___/___  Normal  Abnormal  Not done

 Unknown If abnormal:  Cavitary  Non-cavitary Miliary Pattern

**5) Exposure for current TB diagnosis: (Attach Contact Investigation Summary Form to back of this form)**

5.1 **Was contact investigation initiated?**  Yes  No

**If yes ,**

5.2 . **Any documented potential sources of infection?**  Yes  No

If yes: Name Location:________________________

Relationship

Date of possible exposure _____ / ______ (MM/YY) to ______ / ________ (MM/YY)

Name Location:

Relationship

Date of possible exposure _____ / ______(MM/YY) to ______ / ________(MM/YY)

5.3. **Any documented potential secondary TB cases?**  Yes  No

**If yes:** Name Location:

Relationship

Date of possible exposure _____ / ______ to ______ / ________

Name Location:

Relationship

Date of possible exposure _____ / ______ to ______ / ________

**6 ) High risk locations**

6.1**. Homelessness in past 3 years:**  Yes  No  Unknown

6.1a. Stayed in homeless shelter:  Yes  No  Unknown

If yes; where (start with most recent shelter) *If more than 2, write on back of page*

Shelter Name (MM/YY – MM/YY))

City State Dates of stay ______-______

Shelter Name (MM/YY – MM/YY))

City State Dates of stay ______-______

6.2**. Hospitalizations in past 3 years**  Yes  No  Unknown

Facility Name

City State Date of admission _____/_______(MM/YY) Date of discharge ______/_______(MM/YY)

Facility Name

City State Date of admission ____/_____(MM/YY)

Date of discharge ____/____(MM/YY)

Facility Name __________________

City State Date of admission ______/_______(MM/YY) Date of discharge ______/_______(MM/YY)

6.3. **Nursing Home Residence in past 3 years**  Yes  No  Unknown

Facility Name

City State Date of admission ___/___ (MM/YY)

6.4 **Incarcerations past 3 years**  Yes  No  Unknown

Facility Name

City State Date of admission _____/_____(MM/YY) Date of discharge _____/_____(MM/YY)

Facility Name

City State Date of admission _____/_____(MM/YY) Date of discharge _____/_____(MM/YY)

6.5 **Are any other location(s) that may be of interest noted in record?**  Yes  No  Unknown

Place/Facility Name

City State Dates___ / (MM/YY) to MM/YY)

Place/Facility Name

City State Dates___ / (MM/YY) to MM/YY)

**7. Comments**

Narrative (Anything else of value from chart that you feel would be useful for retracing patient during their time of infectiousness, locations, or participation in activities with other people that potentially placed them at risk for transmission?) *(continue on back if needed)* ______________________________________________________________________________________

___________________________________________________________________________________________________

__________________________________________________________________________________________________

**Step 4: Cluster investigation TB patient interview**

*INSTRUCTIONS: Please note that this form is designed to be used as a guide to probe for relationships among MTB clustered patients, or for places where TB transmission may have occurred, much like a contact investigation is done. The form is not meant to be a structured interview tool. Developing a relaxed, open, and trusting relationship between the patient and you is essential. Use your best judgment to ask the patient each question. Watch for commonalities between this patient and other patients in the MTB genotype cluster.*

RVCT# ___________________ Cluster designation date: ________________________________

Date of Interview: ______/______/______ (*MM/DD/YY)* Location of Interview: Home Clinic Other:____________________

Interviewer initials: TB provider’s name:________________________________

**1. Information to contact patient**

1.1. **Do you have another name such as a nickname, alias, or maiden name?**  **Yes No Refused**

If **Yes,** *List names*) _______________________________________________________________________________________

1.2. **What is your current address?** _________________________________________________________________________ _______________________________________________________________________________________________________

1.3. **In case we need to contact you again, what is the phone number where we can reach you?**

Cell: _________________ Home: ____________________ Work: ___________________

1.4. **Is there another person we can contact in case we can’t reach you?** **Yes No**  **Refused**

If **Yes,** Name ___________________________________ Telephone number(s):_______________________

**2. TB history**

2.1. **What was your first symptom that led to your TB diagnosis?** *(list)____________________________*

2.1a. When did this symptom begin? __________ (*MM/YYYY)*

2.2. **Did you have a cough before you were told you had TB?**

**Yes No Don't know Refused**

2.2a. If **Yes**, when did you start coughing? (*MM/YYYY*)

**2.2b.** If **Yes,** did you cough up blood?

**Yes No Don't know Refused**

2.3. **Had you ever been sick with TB before this time**? **Yes No Don't know Refused**

If **Yes**, where and when? City: State:_____ Country:___________

Date (*MM/DD/YYYY*): __________________

2.4. **Did you ever have skin or blood tests for TB**? **Yes No Don't know Refused**

2.4.a. If **Yes**, ever have a positive test? **Yes No Don't know Refused**

2.4b. If **Yes**, date of first positive test? _______________ *(MM/YYYY)*

2.4c. Date of last negative test? _______________ *(MM/YYYY)*

2.5. **When were you told you had TB**? ______________(*MM/YYYY→MM/YYYY*)

2.6. **How do you think you got sick with TB?** (*If person is mentioned, obtain name, relationship, & time of possible exposure)*

__________________________________________________________________________________________________________________________________________________________________________________

2.7. **After you got TB (this diagnosis), did anybody around you get sick with TB?**

**Yes No Don't know Refused**

2.7a. If **Yes**, who? _____________________ 2.5d. If **Yes**, who? _____________________

2.7b. **What was their relationship to you?** 2.5e. **What was their relationship to you?**

**Parent Grandparent Spouse Child Parent Grandparent Spouse Child**

**Sibling Other relative Coworker Friend Sibling Other relative Coworker Friend**

**Other** (*Specify*) **Other (***Specify*)

2.7c. **Where? City** 2.5f. **Where? City**_____________________

**State Country _____________ State ______ Country*______________***

Additional Information: _________________________________________________________________

_____________________________________________________________________________________________

2.8. **At any time in your life, did you know anybody who had a severe cough, coughed for a long time, coughed up blood, and lost a lot of weight?** **Yes No Don't know Refused**

2.8a. If **yes,** how many people did you know with these severe symptoms? *Complete for ALL below:*

2.8b **Name #1**

2.8c. What was their relationship to you?  **Parent Grandparent Spouse Child Sibling**

**Other relative Coworker Friend Other (*Specify*)**

2.8d. Were you around that person **when they were coughing**? **Yes No Don't know Refused**

2.8e. When and for how long? (*MM/YYYY→MM/YYYY*)

2.8f. Where? City State Country ______________ Zip code_____________

2.8g. **Name#2**

2.8h. What was their relationship to you?  **Parent Grandparent Spouse Child Sibling**

**Other relative Coworker Friend Other (*Specify*)**

2.8i. Were you around that person **when they were coughing**? **Yes No Don't know Refused**

2.8j. If **Yes**, when and for how long? (*MM/YYYY→MM/YYYY*)

2.8k Where? City State Country ______________ Zip code___________

*(Complete additional persons on back or add another page to form)*

2.9. **At anytime in your life, did you know anybody who had TB?**  **Yes No Don't know Refused**

2.9a. How many people did you know with TB?_________ *Please add information for ALL below:*

2.9b **Name #1?**

2.9c. What was their relationship to you? **Parent Grandparent Spouse Child Sibling**

**Other relative Coworker Friend Other (*Specify*)**

2.9d Were you ever around that person **when they were sick with TB**? **Yes No Don't know Refused**

2.9e. If **Yes**, when and for how long? (*MM/YYYY→MM/YYYY*)

2.9f. Where? City State Country _________ Zip code: __________

2.9g**. Name #2?**

2.9h. What was their relationship to you? **Parent Grandparent Spouse Child Sibling**

**Other relative Coworker Friend Other (*Specify*)**

2.9i. Were you ever around that person **when they were sick with TB**? **Yes No Don't know Refused**

2.9j. If **Yes**, when and for how long? (*MM/YYYY→MM/YYYY*)

2.9k. Where? City State Country __________ Zip code: __________

*(Complete additional persons on back or add another page to form)*

**3. Medical Risk**

3.1. **Have you ever been told that you are HIV positive or have HIV or AIDS? Yes No Don't know Refused**

3.2. **Have you ever been told you have medical conditions such diabetes, rheumatoid arthritis,**

**dialysis for kidney disease, or cancer?** **Yes No Don't know Refused**

3.2. **Are you currently taken medications such as steroids or chemotherapy?** **Yes No Don't know Refused**

3.3. **Regular visit to clinic or hospital for treatment for immunosuppressive condition**: **Yes No Don't know Refused**  If **yes**: name of hospital or clinic: _________________Location: _____________________________

**4. Locations**

**In this part of the interview, we want to see if we can find the place and time you were exposed to someone with TB. I will start by asking about places you have lived 3 years before you were diagnosed with TB. Also, I will ask of indoor places you would go often, for example workplace, schools, church, or social meeting places such as clubs, bars, and restaurants**.

**You were diagnosed with TB on _________** (*MM/DD/YYYY* *from Patient tracking form*).

**Think back 3 years from this date.**

(*Provide approximate date ______________. Use holidays to help focus on time)*

4.1. Where have you lived during the past 3 years before your TB and how long did you live at each place? (*MM/YYYY→MM/YYYY*)

City/town State Zip Code Country Dates___________

City/town State Zip Code Country Dates___________

City/town State Zip Code Country Dates___________

4.2. During the 3 years prior to your TB, have you traveled outside of the U.S.? **Yes No Don’t Know Refused**

If **yes,** Where and when did you go?

(*MM/YYYY→MM/YYYY*)

City/town State Zip Code Country Dates___________

City/town State Zip Code Country Dates___________ City/town State Zip Code Country Dates___________

4.3. During the 3 years prior to your TB, have you had any visitors (relatives or friends) who

traveled from another country to the U.S. ? **Yes No Don’t Know Refused**

4.4. If you attended school during the past 3 years, please give school names and dates.

(*MM/YYYY→MM/YYYY)*

Name#1 Location Dates

Name#2 Location Dates

4.5 Where did you work in the past 3 years? Please give workplace name and dates.

Name#1 Location: :

Dates .(*MM/YYYY→MM/YYYY)*

Name#2 Location: :

Dates .(*MM/YYYY→MM/YYYY)*

Name#3 Location: :

Dates .(*MM/YYYY→MM/YYYY)*

4.6. If you had a place of worship, please give their names, how often you attended, and what time period. (*MM/YYYY→MM/YYYY*)

Name Location: Dates

**Frequencies: ≥ 1/week 1/week 1/month <1/month**

Name Location: Dates

**Frequencies: ≥ 1/week 1/week 1/month <1/month**

Name Location: Dates

**Frequencies: ≥ 1/week 1/week 1/month <1/month**

- 1. In the past 3 years, do you regularly socialize in the evenings or other with other people to play cards, talk, etc.

*(Examples might be friends’ homes, social clubs, bars, restaurants, … places to share drugs)*

4.7a. Place name: _________________________ Location ____________________________

Activity: ________________________________________________

Type of establishment **Social Club Bar Restaurant Community Center Other** (*Specify*):__________________________________________

4.7b. Place name: ________________________ Location: ____________________________

Activity: ________________________________________________

Type of establishment **Social Club Bar Restaurant Community Center Other** (*Specify*):____________________________________________

4.8 In the past 3 years, during the daytime hours *(such as ‘When you get up’, ‘go to work’ ,‘at lunch’, ‘come home’),* where do you usually spend time with other people*? (Examples might be Elks, Masons, VFW, Junior League, senior citizens centers, day care centers, video arcades, bars, restaurants, or social clubs)*

4.8a. Place name:_________________________ Location ____________________________

Activity: ________________________________________________

Type of establishment **Social Club Bar Restaurant Community Center Other** (*Specify*):____________________________________________

4.8b. Place name:_________________________ Location: ____________________________

Activity: ________________________________________________

Type of establishment ( **Social Club Bar Restaurant Community Center**

**Other** (*Specify*):_________________________________

4.8c. Place name:_________________________ Location: ____________________________

Activity: ________________________________________________

Type of establishment **Social Club Bar Restaurant Community Center Other** (*Specify*):_____________________________________

4.9. In the past 3 years, have you **worked** in a hospital, clinic, nursing home, drug treatment center, detoxification center, shelter, detention center, prison, rescue mission, or Salvation Army?

**Yes No Don't know Refused**

4.9a. If **Yes**, give name of the facility, its location and dates you worked there.

Name/City/State/Country, if applicable Dates *(MM/YYYY→MM/YYYY)*

4.9b. What type of facility was it? (*Circle one*) **Hospital Clinic Nursing Home Shelter**

**Drug treatment center Other** (*Specify*)

(*Use back of sheet to record further information on above question*)

4.10. In the past 3 years, have you been in the military or National Guard*?* **Yes No Don't know Refused**

4.10a**.** If **Yes,** what service were you in? (***Circle one*)**

**Army Navy Air force Marines National Guard**

**Coast Guard Other (***Specify*)___________________________________________

4.10b. Where were you stationed? Please give dates for each location.

Name/State/Country Dates (*MM/YYYY→MM/YYYY*)

4.11. In the past 3 years, have you stayed in a dormitory for school, college or other non-military settings?

(*Circle one*) **Yes No Don't Know Refused**

4.11a. If **Yes**, please give the place and dates.

Name/State/Country Dates (*MM/YYYY→MM/YYYY*)

4.12. In the past 3 years, have you stayed in a refugee camp? **Yes No Don't Know Refused**

4.12a. If **Yes**, please give the place and dates.

City Country Dates *(MM/YYYY→MM/YYYY)*

4.13. In the past 3 years, have you stayed in a nursing home or group living home? (*Includes halfway houses, orphanages, foster homes, etc.*) **Yes No Don't know Refused**

4.13a If **Yes**, please list the place and time.

Name/City/State Dates *(MM/YYYY→MM/YYYY)*

4.14. In the past 3 years, have you been in a detention center, jail, or prison?

**Yes No Don't know Refused**

4.14a If **Yes**, please list the place and time.

Name/City/State Dates *(MM/YYYY→MM/YYYY)*

4.15. In the past 3 years, have you been a member of a daytime activity group, day care, or senior center

**Yes No Don't know Refused**

4.15a. If **Yes**, please list the name, location and dates for each

Name/City/State Dates *(MM/YYYY→MM/YYYY)*

4.16. In the past 3 years, have you ever lived on the streets or been homeless? **Yes No Don't know Refused**

4.16a. If Y**es**, how long _____________________________________________ (Months / Years)

4.17 . In the past 3 years, have you stayed in a shelter, rescue mission, Salvation Army, or lived in a hotel?

**Yes No Don't know Refused**

4.17a. If **Yes**, please list the name and location of each place and dates you stayed there.

Name of place/City/State Dates *(MM/YYYY→MM/YYYY*)

4.18. In the past 3 years, have you stayed in a hospital? **Yes No Don't know Refused**

4.18a. If **Yes**, please list the hospitals and the dates you stayed in each.

Name of Hospital/City/State Dates *(MM/YYYY→MM/YYYY)*

_______________

4.19. In the past 3 years, have you been treated at a drug treatment or alcohol detoxification (detox) center?

(*Circle one*) **Yes No Don't know Refused**

4.19a. If **Yes**, please list the place and time you were treated in each one.

Name of Facility/City/State Dates (MM/YYYY*→*MM/YYYY)

**This is the end of the interview. Can you think of anything else that may be helpful?**

_______________________________________________________________________________

_______________________________________________________________________________

_______________________________________________________________________________

**Thank you for taking the time to talk with me.**
